# Virulence genes of *Rickettsia rickettsii* are differentially modulated by either temperature upshift or blood-feeding in tick midgut and salivary glands

**DOI:** 10.1186/s13071-016-1581-7

**Published:** 2016-06-10

**Authors:** Maria Fernanda B. M. Galletti, André Fujita, Rafael D. Rosa, Larissa A. Martins, Herbert S. Soares, Marcelo B. Labruna, Sirlei Daffre, Andréa C. Fogaça

**Affiliations:** Departamento de Parasitologia, Instituto de Ciências Biomédicas, Universidade de São Paulo, São Paulo, Brazil; Departamento de Ciências da Computação, Instituto de Matemática e Estatística, Universidade de São Paulo, São Paulo, Brazil; Departamento de Medicina Veterinária Preventiva e Saúde Animal, Faculdade de Medicina Veterinária e Zootecnia, Universidade de São Paulo, São Paulo, Brazil; Departamento de Biologia Celular, Embriologia e Genética, Universidade Federal de Santa Catarina, Santa Catarina, Brazil

**Keywords:** *Rickettsia*, Virulence, Tick, Midgut, Salivary glands, Transcription

## Abstract

**Background:**

*Rickettsia rickettsii*, the etiological agent of Rocky Mountain spotted fever, is transmitted to humans by ticks. During tick feeding, *R. rickettsii* is exposed to both temperature elevation and components of the blood meal, which have previously been associated with the reactivation of its virulence. These environmental stimuli were also reported to modulate virulence genes of *R. rickettsii* infecting a set of organs of adult females of its natural vector, *Amblyomma aureolatum*.

**Methods:**

In this study, we determined the effects of a temperature upshift, blood-feeding, and both stimuli simultaneously on the expression of 85 selected genes of *R. rickettsii* infecting either the midgut (MG) or salivary glands (SG) of male and female *A. aureolatum* by microfluidic high-throughput RT-qPCR. These two organs are key for acquisition of this bacterium by the tick and transmission to the vertebrate host, respectively.

**Results:**

Data showed that these environmental stimuli exert distinct effects on rickettsial transcription depending on the colonized organ and gender of the vector. Temperature upshift induced the majority of differentially expressed genes of *R. rickettsii* in tick SG, including tRNA synthetases encoding genes. On the contrary, blood-feeding downregulated most of differentially expressed genes in both organs, but induced type IV secretion system components and OmpB in tick MG. The combined effects of both stimuli resulted in a merged gene expression profile representing features of each stimulus analyzed independently, but was more similar to the profile induced by blood-feeding.

**Conclusion:**

The upregulation of the majority of differentially expressed genes in tick SG by temperature upshift suggests that this stimulus is important to prepare *R. rickettsii* for transmission to the vertebrate host. Blood-feeding, on the other hand, induced important virulence genes in the tick MG, which might be associated with colonization of the tick and transmission to the vertebrate host. The role of the proteins identified in this study must be addressed and might help to define future targets to block tick infection, thereby preventing RMSF. To our knowledge, this is the first transcriptional tissue-specific study of a virulent strain of *R. rickettsii* infecting a natural tick vector.

**Electronic supplementary material:**

The online version of this article (doi:10.1186/s13071-016-1581-7) contains supplementary material, which is available to authorized users.

## Background

The tick-transmitted pathogen *Rickettsia rickettsii* (Rickettsiales: Rickettsiaceae) is the etiological agent of the most lethal spotted fever rickettsiosis that affects humans, Rocky Mountain spotted fever (RMSF). Infection of endothelial cells by *R. rickettsii* causes an intense vasculitis, which can lead to the failure of important organs, including the brain, lungs, and kidneys [1–3]. Although antibiotic treatment is available, it is only effective in the early stages of the disease [3]. Furthermore, the non-specific nature of clinical manifestations, such as fever, headache, and myalgia along with the late detection of antibodies to *R. rickettsii* in serological tests, makes early diagnosis difficult [3]. For this reason, fatality rates of RMSF are high, reaching approximately 40 % in Brazil [4]. RMSF is also considered a reemerging disease, with a rise in the number of cases in recent years [3; 4]. Notably, the annual incidence increased dramatically in the United States from one to over nine cases per million people from 2000 to 2010 [5]. Given the absence of a vaccine, prevention still relies on avoiding contact with *R. rickettsii-*infected vectors [6].

When a tick encounters a host, it inserts its mouthparts into the host skin and begins blood acquisition. If the host is infected, bacteria initially acquired within the blood meal must colonize the midgut (MG) and later the salivary glands (SG) through hemolymph. During a successive blood-feeding, these microorganisms are transmitted to the vertebrate host via saliva [7]. The presence of *R. rickettsii* in hemocytes of experimentally-infected *Rhipicephalus sanguineus* (*sensu lato*) (*s.l*.) suggests that these cells might play a role in spreading rickettsiae throughout the tick organs [8]. The epithelium of tick MG is largely composed of endocytic cells responsible for the intracellular digestion of proteins contained in the ingested blood meal [9]. Conversely, the cells of SG acini are highly secretory, as these organs produce saliva. Tick saliva, which is inoculated into the host during blood acquisition, contains an arsenal of anti-hemostatic, anti-inflammatory, and immunomodulatory molecules, assuring acquisition of blood and simultaneously facilitating pathogen transmission [10, 11]. Due to the different physiologic functions of tick MG and SG, these two organs also express different sets of genes, including those involved with tick immune reactions [7]. Therefore, to be able to adapt to such distinct niches, the bacteria may significantly alter their transcription machinery in each organ.

In addition to the different niches that arthropod-borne pathogens are obliged to colonize in order to ensure successful transmittion to the vertebrate host, they must also deal with the different immune responses that may be exerted depending on the sex of the vector. For instance, females of *Drosophila melanogaster* were reportedly more sensitive than males to an infection with the fungus *Beauveria bassiana* [12]. Conversely, male house crickets, *Acheta domesticus*, were more susceptible to experimental infection with *Serratia liquefaciens* [13]*,* whereas no differences in response to a microbial challenge were observed between genders of the moth *Galleria mellonella* [14].

We have previously reported the global gene expression profile of *R. rickettsii* infecting a complete set of internal organs of adult females of its natural tick vector, *Amblyomma aureolatum*, exposed to either a temperature upshift of 10 °C or blood-feeding [15]. Importantly, those two environmental stimuli have been associated with reactivation of rickettsial virulence in ticks [16–19]. The increase in temperature caused a limited transcriptional modulation in *R. rickettsii* [15], as also observed in a tick cell line in vitro [20]. However, the exposure to the blood meal modulated approximately five times more rickettsial genes [15]. In the current study, we demonstrate the effects of temperature upshift, blood-feeding or both stimuli simultaneously, on the transcriptional profile of selected genes of *R. rickettsii* infecting two specific organs, SG and MG, of *A. aureolatum* males and females.

## Methods

### *Rickettsia rickettsii* and *Amblyomma aureolatum*

The virulent Taiaçu strain of *R. rickettsii* was originally isolated from a naturally infected *A. aureolatum* tick [21]. Since its original isolation, this strain has been cryopreserved in the organs of infected guinea pigs, with no in vitro passage. An *A. aureolatum* laboratory colony 100 % infected with *R. rickettsii* was generated as previously described [15, 22].

### Exposure to environmental stimuli

A group of *R. rickettsii*-infected unfed adult females and males were incubated at either 25 °C (Group 1, G1) or 35 °C (Group 2, G2) for 3 days or fed on domestic dogs (*Canis lupus familiaris*) for 3 days (Group 3, G3). Canine infestations were performed as previously described [15, 22]. Immediately after incubation or feeding, the ticks were washed in 70 % ethanol and subsequently in sterile phosphate-buffered saline (PBS) (10 mM NaH_2_PO_4_, 1.8 mM KH_2_PO_4_, 140 mM NaCl, and 2.7 mM KCl, pH 7.4) for 10 min each. To collect organs from individual ticks, the cuticle was carefully cut and removed. Midgut (MG) and salivary glands (SG) were separated, transferred to 50 μl RNA*later*^*®*^ Solution (Thermo Scientific, Walktham, USA) and maintained at -20 °C until further processing.

### Nucleic acid isolation

Simultaneous isolation of genomic DNA (gDNA) and total RNA from the organs of each individual tick was carried out using the InviTrap^®^ TwinSpin Cell Mini Kit (STRATEC, Birkenfeld, Germany) according to the manufacturer’s instructions.

### Real-time quantitative PCR (qPCR)

To determine the total number of rickettsiae per each tissue, absolute quantification by real-time qPCR was performed using specific primers and a hydrolysis probe for the citrate synthase gene (*gltA*) as previously described [15, 22, 23].

### Microfluidic high-throughput RT-qPCR

Specific primers for 85 selected *R. rickettsii* genes (Additional file 1: Table S1) were designed using Primer3 [24, 25] and synthesized by Thermo Scientific (Walthan, USA). Among the 85 selected genes, 65 had been previously analyzed by microfluidic high-throughput RT-qPCR during infection of a complete set of internal organs of *A. aureolatum* adult females [15]. Fourteen genes [alkaline protease secretion ATP-binding protein AprD; antitoxin of toxin-antitoxin system StbD; DNA gyrase subunit A; DNA polymerase III subunit beta; nitrogen regulation protein NtrY; ribose-5-phosphate isomerase B; succinate semialdehyde dehydrogenase; tRNA uridine 5-carboxymethylaminomethyl modification enzyme GidA; type IV secretion system component virB4 protein precursor; and five hypothetical protein encoding genes (A1G_01755, A1G_02385, A1G_03005, A1G_03375, A1G_06045)] that had been detected as modulated by microarray experiments in the same referred study as well as 6 genes [thioredoxin; signal peptidase I; and four hypothetical protein encoding genes (A1G_03445, A1G_03570, A1G_04915, A1G_06440)] that have not been modulated, were selected for analysis in the present study. Primers were designed based on the *R. rickettsii* genome [Iowa (NC_010263.2) and Sheila Smith (NC_009882.1) strains] [26].

The total RNA (200 ng) extracted from the MG or SG of ticks harboring 1.08 × 10^8^ ± 3.93 × 10^7^ rickettsiae from each biological group (G1, G2 and G3) was used as template for reverse transcription (RT) to complementary DNA (cDNA), pre-amplified and used in microfluidic high-throughput RT-qPCR as described by [15]. The numbers of biological replicates were 10 males and 10 females for G1 and G2, and 10 males and 9 females for G3. As a non-specific cross-reaction control, tissues from non-infected *A. aureolatum* ticks were equally processed and analyzed.

### Data and statistical analyses

The quantification cycle (Cq) was determined using the BioMark™ qPCR analysis software (Fluidigm, San Francisco, USA), as previously detailed [15]. Threshold values were normalized according to the Cq of three reference genes: ribonuclease H (A1G_06145), outer membrane assembly protein (A1G_02675) and stage 0 sporulation protein J (A1G_00545). The 2^-ΔΔCq^ equation [27] was used to determine the relative expression of genes in the MG and SG of ticks from G2 *versus* G1 (to evaluate the effect of temperature upshift), G3 *versus* G2 (to evaluate the effect of blood-feeding), and G3 *versus* G1 (to evaluate the combined effects of temperature upshift and blood-feeding). Only genes with fold-changes ≥ 1.5 or ≤ -1.5 were considered. To identify differentially expressed genes between the groups, we carried out a Wilcoxon test. *P*-values < 0.05 after correction for multiple tests by the False Discovery Rate procedure [28] were considered statistically significant (differentially expressed between groups). The nomenclature described here is based on the rules of the Minimum Information for publication of Quantitative real-time PCR Experiments (MIQE) [29].

## Results

**Fig. 1:**
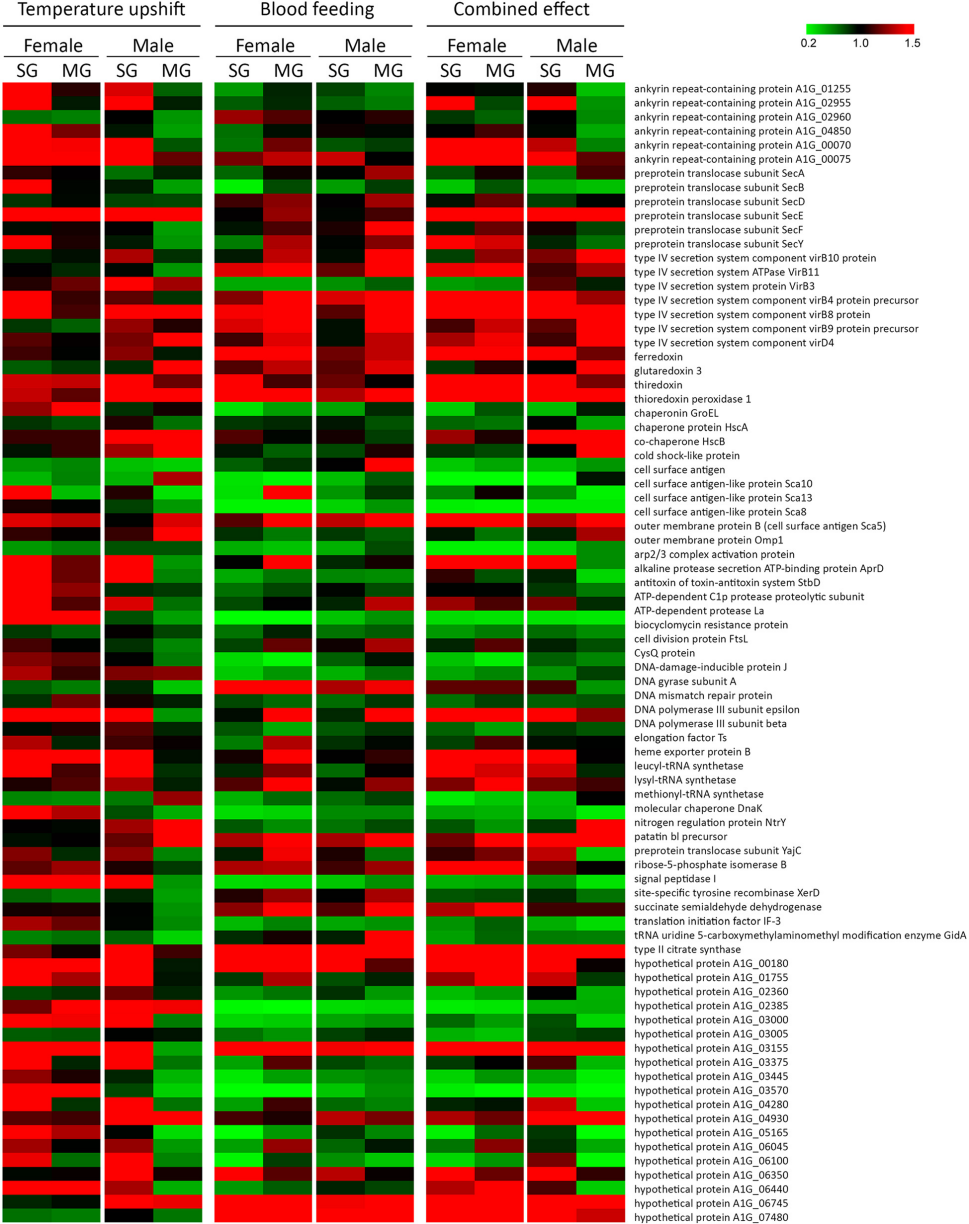
Transcriptional profile of *Rickettsia rickettsii* exposed to temperature upshift, blood-feeding or both stimuli simultaneously. The 2^-ΔΔCq^ equation was used to determine the relative expression of rickettsial genes in the MG and SG of male and female ticks from G2 *versus* G1 (to evaluate the effect of temperature upshift), G3 *versus* G2 (to evaluate the effect of blood-feeding), and G3 *versus* G1 (to evaluate the combined effects of temperature upshift and blood-feeding). Each cell in the matrix corresponds to the expression level of one gene in a sample (mean values of biological replicates). The intensity of the color from green to red indicates the magnitude of differential expression

The comparison of the transcriptional level of *R. rickettsii* genes in either the SG or MG of male and female ticks from G2 *versus* G1 (temperature upshift effects), G3 *versus* G2 (blood-feeding effects) and G3 *versus* G1 (both stimuli simultaneously) revealed that these environmental stimuli exert a potent effect on the gene expression of this bacterium (Fig. 1). Among the 85 genes of *R. rickettsii* that were analyzed, 57 genes were differentially expressed in SG (Table 1 and Additional file 2: Table S2) and 68 in MG (Table 2 and Additional file 3: Table S3) of male and/or female ticks under at least one of these three conditions. The expression of the majority of rickettsial genes was modulated or not in both tick genders (Table 3). In addition, genes that were modulated in both sexes presented the same regulation (up or downregulation) in males and in females (Tables 1, 2; Additional file 2: Table S2; Additional file 3: Table S3). Remaining genes were differentially expressed exclusively in one gender, while the difference in expression in the other was not statistically significant between groups (Tables 1, 2 and 3; Additional file 2: Table S2; Additional file 3: Table S3).

**Table 1:** Transcriptional level of selected genes of *Rickettsia rickettsii* in salivary glands of ticks exposed to temperature upshift, blood-feeding or both stimuli simultaneously. Microfluidic RT-qPCR fold changes (mean of at least nine biological replicates) with statistically significant differences with respect to control are presented

		Temperature upshift				Blood-feeding				Temperature upshift + Blood-feeding			
		Female		Male		Female		Male		Female		Male	
Gene ID	Annotation	Fold change	*W* ^a^ (*P*-value)	Fold change	*W* (*P*-value)	Fold change	*W* (*P*-value)	Fold change	*W* (*P*-value)	Fold change	*W* (*P*-value)	Fold change	*W* (*P*-value)
A1G_00075	Ankyrin repeat-containing protein	ns^b^		ns		ns		ns		2.95	77 (0.0160)	2.39	74 (0.0415)
A1G_01255	Ankyrin repeat-containing protein	ns		ns		-1.92	5 (0.0012)	ns		ns		ns	
A1G_00615	Preprotein translocase subunit SecB	ns		ns		-4.17	0 (0.0001)	-1.96	12 (0.0221)	-2.63	10 (0.0122)	-2.17	14 (0.0277)
A1G_01005	Preprotein translocase subunit SecE	1.54	94 (0.0050)	1.79	100 (0.0009)	ns		ns		1.55	90 (0.0002)	1.73	90 (0.0002)
A1G_04855	Preprotein translocase subunit SecA	ns		-1.54	13 (0.0290)	ns		ns		ns		-1.56	4 (0.0013)
A1G_05455	Preprotein translocase subunit SecY	2.94	94 (0.0050)	ns		-1.64	5 (0.0012)	ns		1.79	75 (0.0242)	ns	
A1G_02210	Type IV secretion system component virB8 protein	ns		ns		ns		ns		2.44	86 (0.0011)	1.96	86 (0.0013)
A1G_06670	Type IV secretion system component virB4 protein precursor	1.69	88 (0.0182)	ns		ns		ns		2.06	84 (0.0021)	1.59	88 (0.0005)
A1G_07055	Ferredoxin	ns		ns		ns		ns		1.61	82 (0.0040)	ns	
A1G_02555	Thioredoxin peroxidase 1	ns		1.57	90 (0.0154)	1.74	90 (0.0001)	ns		2.32	90 (0.0002)	2.04	90 (0.0002)
A1G_00010	Thioredoxin	ns		1.71	97 (0.0021)	ns		ns		1.99	89 (0.0003)	2.01	90 (0.0002)
A1G_01335	Molecular chaperone DnaK	-1.72	11 (0.0143)	ns		-2.22	1 (0.0002)	-1.52	10 (0.0144)	-3.85	0 (0.0002)	-2.44	4 (0.0013)
A1G_01505	Co-chaperone HscB	ns		1.84	99 (0.0009)	ns		ns		ns		1.93	89 (0.0003)
A1G_01545	DNA gyrase subunit A	ns		ns		-2.70	2 (0.0004)	-2.22	14 (0.0332)	-2.08	16 (0.0300)	ns	
A1G_02785	Site-specific tyrosine recombinase XerD	ns		ns		-3.23	0 (0.0001)	-2.63	2 (0.0018)	-2.22	3 (0.0007)	-1.79	8 (0.0052)
A1G_05315	Chaperonin GroEL	ns		ns		-3.45	0 (0.0001)	-2.04	9 (0.0115)	-2.78	6 (0.0021)	-2.44	0 (0.0002)
A1G_07375	DNA-damage-inducible protein J	ns		ns		-3.13	0 (0.0001)	ns		-2.56	3 (0.0007)	ns	
A1G_07470	DNA mismatch repair protein	ns		ns		1.64	87 (0.0006)	1.29	76 (0.0332)	ns		ns	
A1G_00295	Cell surface antigen-like protein Sca10	-2.33	3 (0.0031)	-2.22	9 (0.0123)	-3.45	0 (0.0001)	-2.44	0 (0.0009)	-8.33	0 (0.0002)	-5.56	0 (0.0002)
A1G_01440	Cell surface antigen-like protein Sca8	ns		ns		-4.35	0 (0.0001)	-2.94	0 (0.0009)	-4.00	0 (0.0002)	-3.70	0 (0.0002)
A1G_06915	Cell surface antigen-like protein Sca13	ns		ns		-3.33	9 (0.0047)	ns		ns		ns	
A1G_03790	Cell surface antigen	-1.89	3 (0.0031)	-2.50	5 (0.0034)	ns		ns		-2.70	0 (0.0002)	-2.50	5 (0.0017)
A1G_06030	Outer membrane protein B (cell surface antigen sca5)	ns		ns		ns		ns		1.59	75 (0.0242)	ns	
A1G_02865	Lysyl-tRNA synthetase	1.80	93 (0.0050)	2.02	92 (0.0099)	ns		ns		1.55	78 (0.0122)	ns	
A1G_03300	Leucyl-tRNA synthetase	4.28	96 (0.0036)	2.04	85 (0.0432)	ns		ns		4.51	90 (0.0002)	2.02	76 (0.0277)
RrIowa_1080	Arp2/3 complex activation protein	-1.92	10 (0.0137)	ns		-2.17	0 (0.0001)	-2.22	2 (0.0018)	-4.17	0 (0.0002)	-3.03	5 (0.0017)

^a^ *W* Wilcoxon test statistic (the sum of the ranks of the observations for controls). Let *m* and *n* be the number of observations for controls and treatment, respectively. Then, the *P*-value for the Wilcoxon rank-sum test is given by the number of rank sums lower than the observed rank sum W divided by *m*+*n* choose *n. P*-values were calculated using the R program (www.r-project.org), function *wilcox.test*

^b^ *ns* no significant differences in relation to control; *P* < 0.05 in multiple comparisons by the False Discovery Rate (FDR) method shown

Controls: unfed ticks at 25 °C (G1) for temperature upshift and combined effects of temperature upshift and blood-feeding; unfed ticks at 35 °C (G2) for blood-feeding

**Table 2:** Transcriptional level of selected genes of *Rickettsia rickettsii* in midguts of ticks exposed to temperature upshift, blood-feeding or both stimuli simultaneously. Microfluidic RT-qPCR fold changes (mean of at least nine biological replicates) with statistically significant differences with respect to control are presented

		Temperature upshift				Blood-feeding				Temperature upshift + Blood-feeding			
		Female		Male		Female		Male		Female		Male	
Gene ID	Annotation	Fold change	*W* ^a^ (*P*-value)	Fold change	*W* (*P*-value)	Fold change	*W* (*P*-value)	Fold change	*W* (*P*-value)	Fold change	*W* (*P*-value)	Fold change	*W* (*P*-value)
A1G_00075	Ankyrin repeat-containing protein	ns^b^		ns		ns		ns		1.91	79 (0.0087)	ns	
A1G_01255	Ankyrin repeat-containing protein	ns		ns		ns		-1.72	17 (0.0410)	ns		ns	
A1G_02955	Ankyrin repeat-containing protein	ns		ns		ns		-1.64	14 (0.0244)	ns		ns	
A1G_02960	Ankyrin repeat-containing protein	-1.67	8 (0.0119)	-1.89	13 (0.0228)	ns		ns		ns		-1.75	14 (0.0225)
A1G_04850	Ankyrin repeat-containing protein	ns		ns		ns		ns		ns		-2.13	16 (0.0353)
A1G_00615	Preprotein translocase subunit SecB	ns		-2.00	12 (0.0310)	ns		ns		ns		-2.50	4 (0.0021)
A1G_00890	Preprotein translocase subunit SecF	ns		-1.96	10 (0.0188)	ns		1.54	85 (0.0018)	ns			
A1G_01005	Preprotein translocase subunit SecE	1.51	98 (0.0036)	1.62	85 (0.0037)	ns		ns		1.89	90 (0.0001)	1.82	81 (0.0003)
A1G_05455	Preprotein translocase subunit SecY	ns		-1.89	2 (0.0018)	ns		ns		ns		-1.54	7 (0.0058)
A1G_02210	Type IV secretion system component virB8 protein	ns		ns		2.53	77 (0.0001)	1.61	89 (0.0003)	2.83	89 (0.0002)	2.31	80 (0.0079)
A1G_02225	Type IV secretion system component virB9 protein precursor	ns		ns		1.92	90 (0.0001)	1.62	83 (0.0035)	ns		ns	
A1G_02230	Type IV secretion system component virB10 protein	ns		ns		ns		2.11	90 (0.0002)	ns		1.77	81 (0.0003)
A1G_02235	Type IV secretion system ATPase VirB11	ns		-1.89	2 (0.0018)	2.26	78 (0.0001)	2.38	90 (0.0002)	1.94	89 (0.0002)	ns	
A1G_02240	Type IV secretion system component VirD4	ns		ns		ns		ns		ns		1.89	83 (0.0035)
A1G_06670	Type IV secretion system component virB4 protein precursor	ns		ns		1.97	66 (0.0001)	1.51	82 (0.0048)	2.17	90 (0.0001)	ns	
A1G_02555	Thioredoxin peroxidase 1	ns		ns		1.83	90 (0.0001)	1.50	87 (0.0010)	2.13	90 (0.0001)	2.29	90 (0.0003)
A1G_07055	Ferredoxin	ns		ns		1.74	83 (0.0002)	ns		1.71	88 (0.0003)	ns	
A1G_01335	Molecular chaperone DnaK	-1.79	10 (0.0176)	ns		-1.49	1 (0.0156)	ns		-2.70	0 (0.0001)	ns	
A1G_01500	Chaperone protein HscA	ns		ns		ns		ns		-1.59	7 (0.0023)	-2.08	4 (0.0013)
A1G_01505	Co-chaperone HscB	ns		2.03	95 (0.0024)	ns		ns		ns		1.64	81 (0.0059)
A1G_01545	DNA gyrase subunit A	ns		ns		-1.89	2 (0.0193)	-1.54	16 (0.0344)	-1.72	17 (0.0368)	ns	
A1G_02785	Site-specific tyrosine recombinase XerD	ns		ns		-3.13	0 (0.0012)	-1.82	0 (0.0002)	-2.13	16 (0.0307)	-3.45	0 (0.0003)
A1G_05315	Chaperonin GroEL	ns		ns		-2.00	0 (0.0038)	ns		ns		ns	
A1G_07375	DNA-damage-inducible protein J	ns		ns		-4.55	0 (0.0001)	ns		-4.00	0 (0.0001)	ns	
A1G_07470	DNA mismatch repair protein	-1.64	16 (0.0488)	-2.63	5 (0.0024)	1.91	87 (0.0003)	ns		ns		ns	
A1G_00295	Cell surface antigen-like protein Sca10	ns		ns		-4.55	0 (0.0001)	ns		-7.69	0 (0.0001)	ns	
A1G_01440	Cell surface antigen-like protein Sca8	ns		-1.92	13 (0.0368)	-4.35	0 (0.0001)	-1.89	4 (0.0012)	-4.35	0 (0.0001)	-3.70	0 (0.0003)
A1G_06915	Cell surface antigen-like protein Sca13	ns		ns		2.57	9 (0.0247)	ns		ns		-4.17	15 (0.0477)
A1G_01150	Outer membrane protein Omp1	ns		ns		-1.61	22 (0.0001)	ns		-1.59	0 (0.0001)	ns	
A1G_03790	Cell surface antigen	ns		-2.86	10 (0.0123)	ns		1.51	84 (0.0025)	-2.08	0 (0.0001)	-1.89	9 (0.0059)
A1G_06030	Outer membrane protein B (cell surface antigen sca5)	ns		ns		ns		2.87	90 (0.0002)	1.95	90 (0.0001)	3.93	86 (0.0013)
A1G_02865	Lysyl-tRNA synthetase	1.80	53 (0.0050)	2.02	70 (0.0099)	ns		ns		1.55	58 (0.0122)	ns	
A1G_03490	Succinate semialdehyde dehydrogenase	-1.64	4 (0.0036)	-2.00	5 (0.0024)	ns		ns		ns		-1.56	17 (0.0440)
A1G_07170	Type II citrate synthase	-1.54	12 (0.0236)	-2.94	0 (0.0009)	ns		1.76	14 (0.0002)	ns		-1.67	12 (0.0133)
A1G_05085	Patatin b1 precursor	ns		ns		-1.79	4 (0.0001)	ns		-1.89	0 (0.0001)	ns	
RrIowa_1080	Arp2/3 complex activation protein	-1.67	13 (0.029)	ns		-2.56	0 (0.0001)	ns		-4.17	0 (0.0001)	-1.79	3 (0.0014)

^a^ *W* Wilcoxon test statistic (the sum of the ranks of the observations for controls). Let *m* and *n* be the number of observations for controls and treatment, respectively. Then, the *P*-value for the Wilcoxon rank-sum test is given by the number of rank sums lower than the observed rank sum W divided by *m*+*n* choose *n. P*-values were calculated using the R program (www.r-project.org), function *wilcox.test*

^b^ *ns* no significant differences in relation to control; *P* < 0.05 in multiple comparisons by the False Discovery Rate (FDR) method shown

Controls: unfed ticks at 25 °C (G1) for temperature upshift and combined effects of temperature upshift and blood-feeding; unfed ticks at 35 °C (G2) for blood-feeding

**Table 3:** Comparison of *Rickettsia rickettsii* gene expression in salivary glands and midgut of male and female ticks exposed to temperature upshift, blood-feeding or both stimuli simultaneously.

	Salivary glands		Midgut	
	Genes modulated or not in both genders	Genes exclusively modulated in one gender	Genes modulated or not in both genders	Genes exclusively modulated in one gender
Temperature upshift	40 (05 m and 35 ns)	15	52 (07 m and 45 ns)	16
Blood-feeding	34 (17 m and 17 ns)	21	42 (19 m and 23 ns)	28
Temperature upshift + Blood-feeding	40 (26 m and 14 ns)	16	30 (20 m and 10 ns)	36

m, modulated in relation to control. ns, no significant differences in relation to control

Controls, unfed ticks at 25 °C (G1) for temperature upshift and combined effects of temperature upshift and blood feeding; unfed ticks at 35 °C (G2) for blood feeding

### Temperature upshift

Sixteen rickettsial genes (ten upregulated and six downregulated) were modulated by temperature upshift in female SG and 11 genes (seven up-regulated and four downregulated) in male SG (Table 1; Additional file 2: Table S2; Additional file 3: Table S3). The induction of the majority of modulated genes was triggered only by the elevation in temperature and exclusively in SG (Fig. 1). Among genes induced in both males and females, we highlight the induction of the subunit E of the Sec dependent translocase system (SecE) and two enzymes involved in protein synthesis (leucyl- and lysyl-tRNA synthetases) (Table 1; Additional file 2: Table S2). In females, *secY* was also upregulated, while *secA* was downregulated in males. Genes encoding thioredoxin, thioredoxin peroxidase 1 and co-chaperone HscB were exclusively induced in males, whereas the component of type IV secretion system (T4SS) was induced solely in females.

In tick MG, the increase in temperature modulated a total of 11 genes in females (three upregulated and eight downregulated) and 19 genes (three upregulated and 16 downregulated) in males (Table 2; Additional file 3: Table S3). Six of seven genes that were modulated in both genders were downregulated, including succinate semialdehyde dehydrogenase and type II citrate synthase. As in tick SG, *secE* was induced in MG of both males and females, whereas the expression of *secB*, *secF* and *secY* was downregulated in males. Transcription of the co-chaperone HscB was induced exclusively in males.

### Blood-feeding

Rickettsiae residing in the SG of female ticks feeding on dogs modulated the expression of 37 genes (six upregulated and 31 downregulated), whereas in feeding males, only 19 rickettsial genes (two upregulated and 17 downregulated) were differentially expressed (Table 1; Additional file 2: Table S2). It is notable that this stimulus caused a strong effect on rickettsial gene expression in tick SG, downregulating the majority of analyzed genes (Fig. 1). Indeed, only two genes, which encode hypothetical proteins (A1G_000180 and A1G_07480), were upregulated in males. Four of the six genes that were upregulated in females also encode hypothetical proteins (A1G_00180, A1G_06350, A1G_06745 and A1G_07480), while the other two genes encode thioredoxin peroxidase 1 and DNA mismatch repair protein. In MG, 34 genes (15 upregulated and 19 downregulated) were modulated in females, whereas 31 genes (14 upregulated and 17 downregulated) were modulated in males (Table 2; Additional file 3: Table S3). In contrast to rickettsiae residing in salivary glands, those infecting MG upregulated the expression of several genes in response to acquisition of the blood meal by ticks (Fig. 1). These included components of the T4SS, which were induced in both genders (Table 2; Additional file 3: Table S3). The gene encoding the cell surface antigen 5 (Sca5, also known as OmpB) was induced exclusively in males, whereas *sca13* was induced only in females. *sca8* was downregulated in both males and females and *sca10* and *omp1* (outer membrane protein 1) were downregulated only in females. Thioredoxin peroxidase 1 and ferredoxin encoding genes were upregulated only in MG of females.

### Combined effects of blood-feeding and temperature upshift

Forty rickettsial genes were modulated in female SG (18 upregulated and 22 downregulated) and 29 (13 upregulated and 16 downregulated) in male SG (Table 1; Additional file 2: Table S2) by blood-feeding combined with temperature upshift. In MG, 42 genes were modulated in females (20 upregulated and 22 downregulated) and 39 in males (12 upregulated and 27 downregulated) (Table 2; Additional file 3: Table S3). The combination of blood-feeding and temperature upshift elicited a composite transcription profile containing features induced by each stimulus analyzed independently (Tables 1, 2; Additional file 2: Table S2; Additional file 3: Table S3), although it was more similar to blood-feeding (Fig. 1). For instance, genes encoding components of T4SS and OmpB, which were induced by blood-feeding, were also induced in tick MG by both stimuli simultaneously. Expression of *secE*, induced in both SG and MG of ticks by temperature upshift, was also up-regulated by association of this stimulus with blood-feeding. Other genes were modulated only when temperature upshift was combined with blood-feeding, such as *virB8* and a gene (A1G_00075) encoding an ankyrin repeat containing protein, that were induced in SG of males and females. In addition, expression of the genes encoding the chaperone HscA and the cell division protein FtsL were downregulated in MG of both genders (Tables 1, 2; Additional file 2: Table S2; Additional file 3: Table S3).

## Discussion

The present study presents the effects of a 10 °C increase in temperature, blood-feeding, or both stimuli simultaneously, on the expression of selected genes of *R. rickettsii* infecting either MG or SG of *A. aureolatum* males and females. These two organs are key for colonization of the vector and transmission to the vertebrate host, respectively. Most of the genes selected for the present analysis had been previously detected as modulated by either temperature upshift or blood-feeding during the infection of a complete set of internal organs of *A. aureolatum* adult females [15]. *Rickettsia rickettsii* is naturally exposed to these two environmental stimuli when an infected starving tick attaches to the warm skin of a vertebrate host and begins blood-feeding. Importantly, these stimuli were associated previously with the reactivation of rickettsial virulence within ticks [16–19].

Our data show that the expression of the majority of the genes of *R. rickettsii* analyzed was modulated or not in both male and female ticks from G2 *versus* G1 (temperature upshift effects), G2 *versus* G3 (blood-feeding effects) and G1 *versus* G3 (both stimuli simultaneously). In addition, genes that were modulated in both genders were equally up- or downregulated in males and females. Other genes were differentially expressed in one gender, whereas the expression in the other gender was not statistically significant between groups. The similarity of the transcriptional pattern of *R. rickettsii* in males and females might be associated with the role played by both genders in the transmission of this bacterium to vertebrate hosts [22]. Moreover, it was previously suggested that males might transfer bacteria to females during mating [30], whereas females transmit *R. rickettsii* to the progeny in a process referred to as transovarial transmission [31].

Temperature upshift induced the expression of the majority of genes in tick SG. Indeed, this was the unique stimulus that triggered the upregulation of most of modulated genes in one specific organ. Among the upregulated genes, we highlight those encoding lysyl- and leucyl-tRNA synthetases, which were induced in both males and females. The upregulation of these genes suggests that the elevation in temperature stimulates the synthesis of proteins in SG, even within starving ticks, where energy and nutrients are scarce. We can therefore hypothesize that this stimulus is important to prepare the bacteria within tick SG for transmission to the vertebrate host. On the contrary, the temperature upshift downregulated most of *R. rickettsii* genes in tick MG, including some involved in energy production and conversion, such as succinate semialdehyde dehydrogenase and type II citrate synthase.

The *secE* gene was also induced in SG and MG of male and female ticks by the increase in temperature. SecE, together with SecY and SecG, compose the translocases of the general secretion system [32]. In Gram-negative bacteria, this system is involved in the transport of proteins from the bacterial cytoplasm to the periplasm [32]. In addition, *secE* and *secY* were also induced in SG of female ticks. The expression of *secE* was also upregulated by temperature upshift in combination with blood-feeding in SG and MG of males and females, as was *yajC*. The latter gene encodes the preprotein translocase subunit YajC, which is an auxiliary subunit of the general secretion system in *Escherichia coli* [33].

The increase in temperature downregulated gene expression of the chaperone DnaK in both SG and MG of females. In addition, other analyzed chaperones were not significantly modulated by this stimulus in females. The same result has been previously detected in *R. rickettsii* infecting the complete set of internal organs of adult females [15], suggesting that a temperature upshift of 10 °C does not trigger a bacterial heat-shock response in this gender. Conversely, the co-chaperone HscB was induced in both SG and MG of males. The expression of thioredoxin and thioredoxin peroxidase 1 encoding genes was also upregulated by temperature upshift in male SG. It is possible that, unlike in females, an elevated temperature triggers an oxidative stress in males, inducing the expression of cell rescue proteins. Indeed, it was previously reported that heat-shock triggers an oxidative stress response in the yeast *Saccharomyces cerevisiae*, upregulating protective proteins, such as heat-shock proteins, thioredoxin, and thioredoxin peroxidase [34]. Thioredoxin peroxidase 1 and ferredoxin encoding genes were also upregulated in MG of females by blood-feeding. On the contrary, some genes associated with recombination and DNA repair (DNA gyrase subunit A, site-specific tyrosine recombinase XerD and DNA-damage-inducible protein J) were downregulated. It is known that the heme component of the blood meal contributes to the formation of free radicals, such as reactive oxygen species (ROS), in blood-feeding arthropods [35]. In addition, it was previously reported that *R. rickettsii* infection induces a pro-oxidant response in human endothelial cells [36, 37], causing oxidative cell injury [38]. Importantly, bacterial DNA has been suggested to be the primary target of ROS when free iron concentrations are high [39]. Therefore, the thioredoxin system might play an important role in protecting *R. rickettsii* from free radicals, especially in fed ticks, in which rickettsial genes involved in DNA repair are downregulated.

Unlike temperature upshift, blood-feeding downregulated most of *R. rickettsii* differentially expressed genes in tick SG and MG. However, the later stimulus induced the expression of some important virulence genes in MG of both genders, including *virB8*, *virB9*, *virB11* and *virB4* that encode components of type IV secretion system (T4SS). In intracellular bacteria, T4SS delivers macromolecules (referred to as effectors) from the bacterium to the host cell, where they interact with different host proteins, enabling bacterial replication and survival [40]. Most effectors of T4SS are ankyrin repeat-containing proteins, known as Anks [41]. For instance, the AnkA of *Anaplasma phagocytophilum* interacts with DNA and nuclear proteins [42], altering the gene transcription of the host eukaryotic cell, especially neutrophils, and favoring microorganism survival [43]. Nonetheless, most of rickettsial genes encoding Anks were downregulated by the environmental stimuli analyzed in the present study. Only one ankyrin encoding gene (A1G_00075) was induced in SG of both genders and in MG of females by temperature upshift and blood-feeding simultaneously. Therefore, identification of the effectors of secretion systems of *R. rickettsii* is warranted so that their role in colonization of the tick and transmission to the vertebrate host may be established. In addition to T4SS components, blood-feeding also upregulated *sca13* in female MG and *sca5* (also known as *ompB*), in male MG. Importantly, OmpB was previously reported to be an important component in the adherence of rickettsiae to host cells [44–46].

As previously mentioned, the combined effects of both environmental stimuli resulted in a composite gene expression profile presenting features of each stimulus analyzed independently, although this profile was more similar to that of blood-feeding. To diminish the effects of intrinsic temperature on G3 samples, we compared the gene expression of *R. rickettsii* within those ticks and unfed ticks exposed to 35 °C (G2). However, the feeding on dog with a body temperature around 40 °C (data not shown) may represent a slight increase in temperature, which might explain the similarity between the transcriptional profiles of both stimuli analyzed simultaneously and blood-feeding alone. In addition to the induction of T4SS components, blood-feeding combined with temperature upshift upregulated *ompB* in MG of both males and females. These results show that blood-feeding alone or in combination with temperature upshift triggers the upregulation of important virulence genes of *R. rickettsii* in tick MG, which might play a role in colonization of the tick and transmission to the vertebrate host. The combination of both stimuli also induced the expression of *secE* and thioredoxin peroxidase 1 encoding gene in both SG and MG of male and female ticks.

## Conclusion

Chronically infected *Amblyomma aureolatum* ticks exposed to a temperature upshift of 10 °C and/or blood-feeding were used to evaluate *Rickettsia rickettsii* gene expression in the MG and SG. To our knowledge, this is the first transcriptional tissue-specific study of a virulent strain of *R. rickettsii* infecting a natural tick vector. Our data demonstrate that these environmental stimuli exert distinct effects on rickettsial transcription depending on the colonized organ and gender of the vector. Our data also show that temperature upshift induces the majority of differentially expressed genes in tick SG, suggesting that this stimulus is important to prepare rickettsiae for transmission to the vertebrate host. In addition, blood-feeding induced important virulence genes in the tick MG, which might be associated with colonization of the tick and transmission to the vertebrate host. The roles that the differentially expressed proteins identified in this study play in tick organs must be addressed; this might help to define future targets to block tick infection and consequently preventing RMSF.

## Additional files

Additional file 1: Table S1. Primers used in RT-qPCR analysis. (XLSX 13 kb) Additional file 2: Table S2. Transcriptional level of *Rickettsia rickettsii* genes in salivary glands of ticks exposed to temperature upshift, blood-feeding or both stimuli simultaneously. (XLSX 16 kb) Additional file 3: Table S3. Transcriptional level of *Rickettsia rickettsii* genes in midgut of ticks exposed to temperature upshift, blood-feeding or both stimuli simultaneously. (XLSX 17 kb)

## Abbreviations

Cq: quantification cycle
MG: midgut
PBS: Phosphate-buffered saline
qPCR: quantitative polymerase chain reaction
RMSF: Rocky Mountain spotted fever
ROS: Reactive oxygen species
RT: Reverse transcription
SG: Salivary glands
T4SS: Type IV secretion system
